# A Year's Progress in Animal Protection

**Published:** 1911-01-21

**Authors:** Ernest Bell

**Affiliations:** Animals' Friend Society, York House, Portugal Street, E.C.


### Editor's Letter-Box.

### A YEAR'S PROGRESS IN ANIMAL PROTECTION.

*To the Editor of* The Hospital.

Sir,—The year which is past is noteworthy for the fact that in it two new laws for the better treatment of animals have been added to the statute-book. Of these two laws, on which the animals are to be congratulated, the first was an extension to Scotland of the Wild Animals in Captivity Act. This Act has worked well in England, and, though its provisions are now hardly up to public opinion, it considerably enlarged the sphere of protection. The second law, known as the Diseases of Animals Act (2), aims at regulating the traffic in worn-out horses. This is a measure of first importance, rendered necessary by the public condemnation of the cruel traffic in question, and is a distinct step in the right direction.

With reference to birds, we may congratulate ourselves on the decision of the Court of Appeal that it is illegal to have in possession any recently caught birds who are protected in the district, though they may have been actually caught in an unprotected area. This is also a step forward, and almost the first one which deals a blow at the cruel practices of bird-catching and bird-caging. An unusual case, brought into court by the R.S.P.C., of blinding a song-bird with a red-hot needle, which was punished by the extreme penalty of the law, also marks an advance in public opinion.

Outside the law courts, the most striking feature of the year's work has been the progress made in the agitation for the better treatment of pit ponies. The question has become recognised by the Press and the public as an important one. Evidence has been taken from a number of witnesses by the Royal Commission on Mines, and we may reasonably hope that some steps will be taken to ameliorate the condition of the helpless sufferers.

On the whole, animal lovers have reason to be pleased with the work of the year, and every step gained gives us a better position for future advance. There is plenty still to do.—Faithfully yours,

Ernest Bell.

Animals' Friend Society,
York House, Portugal Street, E.C.
January 10.

